# Perineural dexamethasone: neurotoxicity or neuroprotection? A systematic review of preclinical evidence

**DOI:** 10.1186/s44158-025-00271-w

**Published:** 2025-08-06

**Authors:** Alessandro De Cassai, Domenico Pietro Santonastaso, Francesco Coppolino, Cristiano D’Errico, Gabriele Melegari, Burhan Dost, Giulia Aviani Fulvio, Annalisa Boscolo, Rafael Boscolo-Berto, Paolo Navalesi

**Affiliations:** 1 Department of Medicine (DIMED), University of Padua, Padua, Italy; 2 Institute of Anesthesia and Intensive Care Unit, University Hospital of Padua, Padua, Italy; 3 Anesthesia and Intensive Care Unit, M.Bufalini Hospital, Cesena, Italy; 4 Department of Woman, Child and General and Specialized Surgery, University of Campania “Luigi Vanvitelli”, Naples, Italy; 5 Anesthesia and Intensive Care Unit, “Nuovo Ospedale della Costiera” Hospital, Sorrento, Italy; 6 Department of Anesthesia and Intensive Care, University Hospital of Modena, Modena, Italy; 7 Department of Anaesthesiology and Reanimation, Ondokuz Mayıs University Faculty of Medicine, Samsun, Türkiye; 8 Department of Cardiac, Thoracic, Vascular Sciences and Public Health, University of Padua, Padua, Italy; 9 Institute of Human Anatomy, Department of Neurosciences, University of Padua, Padua, Italy

**Keywords:** Dexamethasone, Neuroprotective, Neurotoxic, Perineural, Safety

## Abstract

**Background:**

Perineural dexamethasone is widely used as an adjuvant to local anesthetics in regional anesthesia to prolong analgesia. However, concerns persist regarding its potential neurotoxic effects, particularly when administered perineurally. This systematic review aims to synthesize preclinical evidence evaluating the neurotoxicity or neuroprotective properties of perineural dexamethasone.

**Methods:**

A systematic search of PubMed, CENTRAL, Scopus, and Embase was conducted through May 22, 2025. Eligible studies included in vivo or in vitro preclinical models assessing the neurotoxic or neuroprotective effects of perineural dexamethasone compared to control conditions. Risk of bias was assessed using the SYRCLE tool for in vivo studies and a narrative evaluation for in vitro studies. A total of 14 studies (11 in vivo, 3 in vitro) met inclusion criteria.

**Results:**

In vitro studies showed that dexamethasone alone was not neurotoxic at clinically relevant doses but could enhance cytotoxicity when combined with local anesthetics at higher concentrations. In vivo models generally demonstrated no significant long-term nerve inflammation, degeneration or demyelination, with some early protective effects observed in perineural dexamethasone groups. However, all in vivo studies were rated at high risk of bias. In nerve injury models, dexamethasone reduced apoptotic and inflammatory markers when administered immediately post-injury, with limited effect when delayed.

**Conclusions:**

Preclinical evidence supports the general safety of low-dose, preservative-free perineural dexamethasone. Nonetheless, high-dose use, additives, and application in patients with neuropathies may pose risks. Given the high risk of bias in existing studies and minimal added benefit over systemic administration, clinical caution is advised.

**Supplementary Information:**

The online version contains supplementary material available at 10.1186/s44158-025-00271-w.

## Introduction

Nerve blockade is achieved by injecting a local anesthetic (LA) in close proximity to a nerve, thereby blocking voltage-gated sodium channels on the nerve membrane. This inhibition prevents the initiation and propagation of action potentials, ultimately halting nerve impulse transmission and resulting in loss of sensation. Since the early 1990s, anesthesiologists have been investigating potential adjuvants to combine with local anesthetics in regional anesthesia. The aim of using adjuvants is to prolong the duration of analgesia while allowing for lower concentrations of local anesthetics, potentially reducing or eliminating the need for continuous perineural infusion catheters [1]. Among these, dexamethasone—a white, odorless, crystalline synthetic corticosteroid—has been proposed as a valuable adjuvant in regional anesthesia blocks. A recent network meta-analysis involving 100 trials and 5728 patients compared dexamethasone and dexmedetomidine as adjuvants to long-acting local anesthetics, administered either perineurally or intravenously [2]. When the route of administration was not considered, dexamethasone was associated with a longer duration of analgesia and sensory block compared to dexmedetomidine. However, when the intravenous and perineural route compared, no difference was found in both duration of sensory blockade and duration of analgesia [2]. Other meta-analyses align with these findings with evidence remains inconclusive regarding whether perineural administration provides any advantage over systemic administration, as many studies have shown no more than minimal differences in overall analgesic effect between the two routes [3, 4].

It is known that local anesthetics are neurotoxic and may trigger apoptosis through a dose-dependent pattern by impairing the mitochondrial membrane and activating caspase-3 and other products [5].

Additionally, safety concerns have been raised by several preclinical studies suggesting a potential neurotoxic effect of perineurally administered dexamethasone [1]. Despite this evidence, perineural dexamethasone continues to be widely used in clinical practice—so much so that it has been described as a “bad habit that is hard to break.” [6]. Even if the topic is controversial and currently discussed in literature, actual guidelines lack actual recommendations or comments regarding potential dexamethasone neurotoxicity [7, 8].

However, the widespread use of perineural dexamethasone as an adjuvant in regional anesthesia reflects a strong clinical interest in extending analgesia duration while minimizing systemic opioid requirements. Despite its popularity, significant uncertainty persists regarding the safety profile of dexamethasone when administered perineurally. This systematic review was undertaken to address a critical gap in preclinical knowledge: specifically, whether perineural dexamethasone exerts neurotoxic or neuroprotective effects on peripheral nerves. Although several meta-analyses have compared the efficacy of perineural versus systemic dexamethasone, they provide limited insight into its mechanistic effects on neural tissue integrity. Given the absence of definitive guidance in current anesthetic guidelines, a synthesis of preclinical data is essential to inform clinical practice and future research.

The aim of this systematic review was to provide a qualitative synthesis of all preclinical studies that have investigated the potential neurotoxic or neuroprotective effects of perineural dexamethasone.

## Methods

A pre-registered protocol was developed and registered on Open Science Framework (reference: wu92r, registered on 15 May 2025). We followed the PRISMA to improve the overall reporting of this systematic review [9].

### Selection criteria (PICOS)

All research articles involving preclinical animal models (P) that investigated perineural dexamethasone (I) compared to any control intervention (C), while assessing its potential neurotoxic or neuroprotective effects (O), were included in our analysis, with no restrictions applied to the study design (S). Studies evaluating only dexamethasone mixed with other drugs other than local anesthetics were excluded from our analysis.

### Search strategy

#### Electronic searches

A systematic search was performed querying the following database: PubMed, The Cochrane Central Register of Controlled Trials (CENTRAL), Scopus and Embase (accessed via Ovid), from the inception until 22 May 2025. The authors conducted an additional literature search by reviewing the reference lists of both the included articles and relevant systematic reviews to identify any further pertinent studies, a process often referred to as “literature snowballing”. The detailed search strategy for each database is available as Supplementary material (SM) 1.

### Data collection and analysis

#### Study selection

After removing duplicates, each citation was independently screened in a double-blind process based on the title to identify potentially relevant or irrelevant studies. Citations deemed potentially relevant were then reviewed in full-text form. During this phase, each study was classified as “pertinent”, “not pertinent” or “perhaps”. References that received a “not pertinent” judgment from both reviewers were excluded. All remaining manuscripts were then analyzed in full to assess their relevance according to the PICOS criteria of the present systematic review.

#### Data extraction

To ensure systematic and accurate data collection, a standardized data extraction form was developed and pilot-tested by the authors to identify potential issues or ambiguities. Data were then independently extracted by two researchers (AB and BD). In cases of disagreement, a third author (ADC) was consulted to resolve any discrepancies.

### Risk of bias assessment

The risk of bias for each included in-vivo study was independently assessed by two reviewers using the SYRCLE Risk of Bias tool [10, 11], which is specifically designed for preclinical animal studies. The tool evaluates ten domains of bias, including selection, performance, detection, attrition, reporting and other potential sources, with each domain rated as “low”, “high” or “unclear” risk of bias. As the SYRCLE tool does not provide an overall risk of bias judgment, the authors applied predefined criteria to classify studies globally: studies with at least one domain rated as high risk or three or more domains rated as unclear were categorized as having a high risk of bias; studies with all domains rated as low risk were considered low risk of bias; and all other combinations were classified as having an unclear risk of bias. Disagreements between reviewers were resolved through discussion or, when necessary, consultation with a third reviewer.

Regarding the in vitro studies, currently, there is no validated tool specifically designed for evaluating the risk of bias, which presents a significant challenge in ensuring consistent and reliable assessment within this area of research. Due to the lack of standardized instruments, various domains relevant to in vitro experiments—such as study design, methodology, reproducibility and data interpretation—have been narrative evaluated by two researchers blinded to each other and further discussed with a third researcher.

The results of these assessments were summarized and used to inform the interpretation of the review findings.

### Statistical analysis

Descriptive statistics from the included studies were reported as presented in the original articles. Extracted data were synthesized narratively, employing both integrative and aggregative approaches to capture key findings across studies.

## Results

### Study selection and data retrieval

**Fig. 1:**
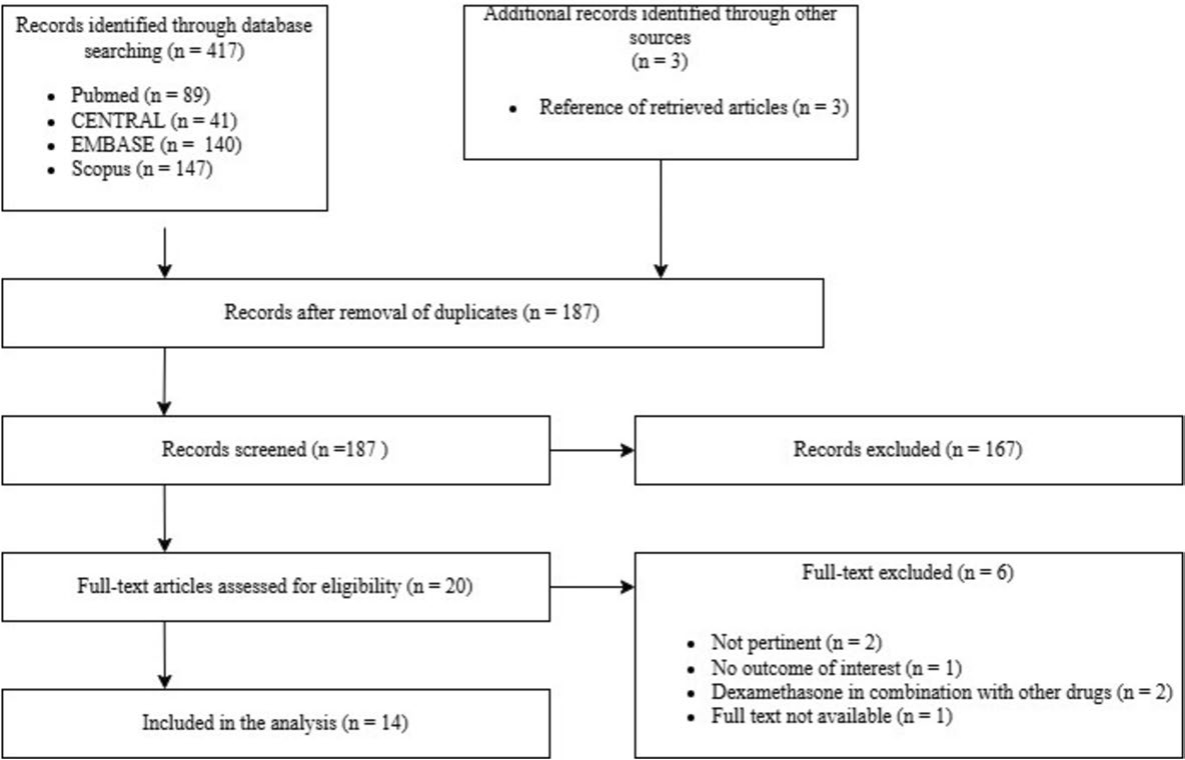
PRISMA flowchart

Search results are shown in the PRISMA flowchart (Fig. 1). Briefly, initial screening identified 417studies. Of these, 230 search results were excluded during the preliminary screening as they were duplicates. The remaining 187 articles were screened by title and abstract, removing a total of 167 as they were not relevant to our search, leaving a total of 20 documents. Further six research were excluded, five research articles after reading the full-text as they do not fully respect our PICOS criteria and one article from 1982 was not available even from the publisher site (details for exclusion available in SDC 1). A total of 14 studies were included in the final analysis [12–25].

### Study characteristics

We included 11 in vivo studies and 3 in vitro studies. The overall characteristics of these studies are summarized in Table 1. The researchers were geographically diverse, representing multiple continents, and nearly all studies reported no conflicts of interest. All in vivo and in vitro studies used mice or mouse-derived cell cultures. With the exception of one study, which focused on the hypoglossal nerve [25], all investigations examined the sciatic nerve.

**Table 1:** Study characteristics

Author	Country	COI	Animal model	Colture/Nerve	Total *N*	Groups	LA	Dexamethasone	Timing	Sham control	Systemic Dexamethasone	Nerve injury model
**In vitro**												
Dani (2007) [12]	Italy	No information	Mice	Glial cell	–	–	B	0.1,1,10,100 µM;P(F,NF)	–	No	–	–
Ma (2010) [13]	China	No information	Mice	Neuroblastoma N2a cells	–	–	B	10, 50,100 µM; P(NS)	–	Yes	–	–
Williams (2011) [14]	USA	None	Sprague–Dawley	Dorsal root ganglion neurons	–	–	R	1291 µM;P(F)	–	No	–	–
In vivo												
An (2015) [15]	USA	None	129S Mice	Sciatic	60	6	B	0.14 mg/kg and 0.5 mg/kg; P(F)	48 h or 7 days	Yes	IM	No
Bastos (2019) [16]	Brazil	None	Male wistar rats	Sciatic	NR	4	No	Perineural implant; P(NS)	NR	Yes	No	Sutured
Çömez (2020) [17]	Turkey	None	Wistar albino rats	Sciatic	54	9	B	0.5 mg/kg; P(NS)	48 h or 7 days	Yes	No	Injection
Ferré (2020) [18]	France	None	C57BL6 wild-type mice	Sciatic	98	7	No	0.15 mg/kg; P(NS)	14 or 28 days	Yes	IP	No
Marty (2018) [19]	France	None	Mice	Sciatic	90	5	R	0.15 mg/kg; P(NS)	14 or 28 days	Yes	IIP	No
Matsuda (2022) [20]	Japan	None	C57BL/6N mice	Sciatic	30	5	R	1.5 mg/kg; P(NS)	< 24 h	Yes	IM	No
Shishido (2002) [21]	Japan	Present	Sprague–Dawley rats	Sciatic	34	3	No	2 mg/kg; P(NS)	2 to 6 days	Yes	No	No
Suslu (2013) [22]	Turkey	No information	Sprague–Dawley rats	Sciatic	32	4	No	2 mg/kg; P(NS)	28 days	Yes	IP	Crush
Wang (2011) [23]	Taiwan	None	Wistar rats	Sciatic	30	4	No	0.8, 1.6, or 3.2; P(NS)	14 days	Yes	No	No
Wang (2015) [24]	Taiwan	None	Wistar rats	Sciatic	32	4	No	0.8 and 3.2 mg; P(NS)	28 days	Yes	No	Sutured
Yao (1995) [25]	Japan	No information	Wistar rats	Hypoglossal	58	2	No	1.5 mg/2w pellet; P(NS)	1 to 42 days	Yes	No	Transection

*B* Bupivacaine, *COI* Conflict of interest, *IM* Intramuscular, *IP* Intraperitoneal, *LA* Local anesthetic, *N* Number, *NR* Not reported, *P*(*F*) Preservatives (free), *P*(*NF*) Preservatives (not free), *P*(*NS*) Preservatives (not stated), *R* Ropivacaine, *w* Week

### Risk of bias

**Fig. 2:**
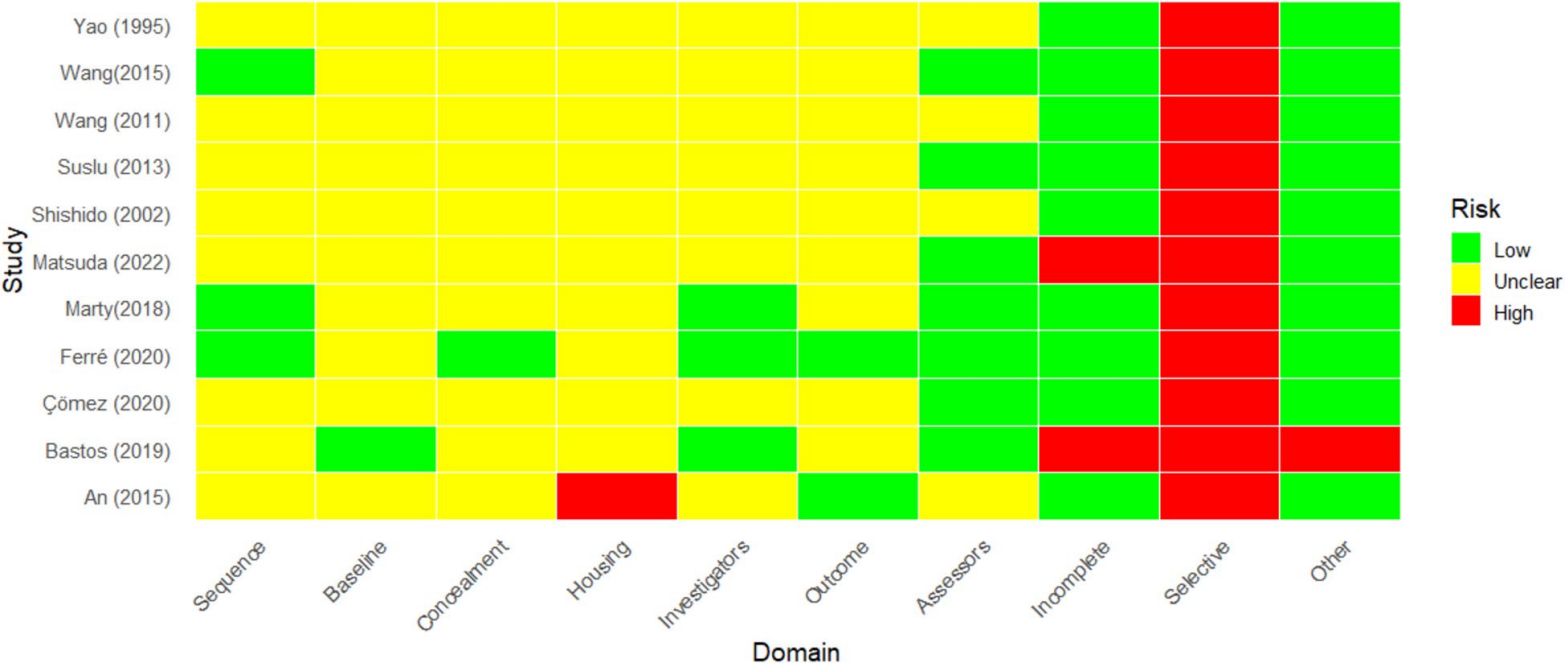
SYRCLE risk of bias assessment

According to our predetermined criteria, all in vivo studies were assessed as high risk, primarily due to the absence of pre-registered protocols and multiple concerns related to the randomization of animals in nearly all studies (Fig. 2). A detailed explanation for the risk of bias assessment is available as SM1. In contrast, the in vitro studies demonstrated reproducibility, and no issues were identified regarding data interpretation. However, some concerns arose from the order in which cell cultures were assigned to different treatments, as well as the lack of blinding of outcome assessors to the treatment groups.

#### In vitro models

Williams et al. [14] harvested dorsal root ganglion neurons to evaluate the neurotoxicity of several adjuvants, both alone and in combination—among them, dexamethasone. The study found that dexamethasone alone was not cytotoxic to neurons after 24 h of exposure. Although the combination of dexamethasone with ropivacaine showed greater neurotoxicity compared to ropivacaine alone (30% vs. 20%), this difference was not reported as statistically significant.

However, the experimental model used by Williams et al. [14] had several limitations that could have led to an overestimation of neurotoxicity. The neurons were already injured prior to isolation, potentially making them more susceptible to additional insults. Additionally, the drug concentrations used were higher than those typically encountered in clinical settings, as the in vitro environment lacks barriers to diffusion and vascular clearance. Furthermore, the study targeted the cell body for drug application, which may be inherently more vulnerable to toxic effects than the distal axons—where drugs are usually administered in vivo.

In another study, Ma et al. [13] demonstrated that pre-exposure to dexamethasone reduced local anesthetic-induced neuronal injury by bupivacaine in a neuroblastoma cell culture model. This protective effect was attributed to the preservation of mitochondrial membrane potential, whose disruption is a known trigger for apoptosis [26]. However, this neuroprotective effect diminished at higher dexamethasone concentrations.

Consistently, Dani et al. [12] reported that dexamethasone at the highest tested concentration (100 µM), whether preservative-free or combined with sulfites, induced significant neural cell death—a response not observed at lower concentrations. Furthermore, the formulations containing sulfites were associated with more pronounced neuronal damage.

#### In vivo models

- No nerve injury models

#### Blood flow

Shishido et al. [21] evaluated sciatic nerve blood flow 30 min after dexamethasone application and observed a substantial decrease of 28.7 ± 19.9% from baseline. A smaller but still significant reduction was also noted in the dorsal root ganglia (14.4 ± 12.1%). However, these decreases were considered insufficient to cause ischemic damage, as the threshold for blood flow reduction associated with nerve ischemia has been estimated in previous studies to be approximately 58% [27].

#### Inflammation

A higher histological rate of nerve inflammation of nerve exposed solely to local anesthetic has been reported at 14 days since the exposition compared to animals exposed to local anesthetic and dexamethasone (in both animals receiving dexamethasone perineurally or systematically); however, the inflammation resulted solved after 28 days since exposition [18]. Results are summarized in Table 2.

**Table 2:** Results of in vivo studies

	Demyelination				Inflammation			
Authors	< 7 d	7 d	14 d	> 21 d	< 7 d	7 d	14 d	> 21 d
An (2015) [15]	+	=						
Ferrè (2020) [18]			=	=			+ ^a^	=
Marty (2018) [19]			=	=			=	=
Shishido (2002) [21]	=				=			
Wang (2011) [23]			=				=	

*+ *Favors perineural dexamethasone, *= *No difference among the animals treated and not treated with dexamethasone ^a^Same results were reported with systemic dexamethasone

*d* Days

All the other studies reporting histological findings reported no difference among the groups.

In particular, Marty et al. [19] observed signs of inflammation in 50% of the nerves at 14 days, with no statistically significant differences among the sham, local anesthetic and dexamethasone groups (both systemic and perineural) and by 28 days, all signs of inflammation had resolved. Similarly, Shishido et al. [21] reported no signs of inflammation at 2, 4 or 6 days, aside from occasional edema. These findings are consistent with those of Wang et al. [23], who found no differences in inflammation at 14 days, although their study lacked detailed data on the degree of inflammation observed.

#### Axon degeneration and myelin regeneration

According to An et al. [15], axon degeneration on day 2 was approximately 6% in mice receiving perineural local anesthetic (with or without systemic dexamethasone), accompanied by reduced S100 expression—a biomarker of myelinating Schwann cell regeneration. In contrast, mice treated with perineural dexamethasone showed only about 3% axon degeneration and higher S100 expression at the same time point. By day 7, however, all groups exhibited similar levels of axon degeneration (around 3%) and S100 expression.

Moreover, no significant caspase expression was detected in the sciatic nerve axoplasm of animals treated with local anesthetic, regardless of dexamethasone administration. Increased nuclear staining consistent with pyknosis—an indicator of cellular stress or early apoptosis—was observed on day 2 in groups treated with local anesthetic (with or without systemic dexamethasone), but not in those receiving perineural dexamethasone. Notably, these apoptotic signs were resolved by day 7 in all groups.

In contrast, other studies reported no differences in axon degeneration between animals treated with perineural dexamethasone and controls during the first week [21], at 14 days [18, 19, 23] and at 28 days [18, 19].

(b) Nerve injury models

It has been hypothesized that dexamethasone may play a protective role in reducing nerve inflammation following injury caused by intraneural injections. To explore this, Çömez et al. [17]. studied a nerve injury model induced by intraneural injection of either saline or bupivacaine. As expected, both saline and bupivacaine groups, regardless of dexamethasone treatment, showed histological and immunohistochemical signs of nerve injury compared to controls. Histologically, all experimental groups exhibited increased edema, vacuolization, myelin degeneration and lymphocytic infiltration, indicating that dexamethasone did not prevent the structural damage associated with nerve injury.

Immunohistochemical analysis revealed that caspase-3 expression—a marker of apoptosis in Schwann cells—was significantly elevated in the intraneural bupivacaine group but reduced in all other experimental groups. Conversely, S100 protein expression was nearly suppressed in the bupivacaine group but preserved in the other groups. Notably, both caspase-3 and S100 levels differed significantly from controls across all experimental groups, suggesting that nerve injury impacted both apoptotic and regenerative cellular pathways regardless of treatment.

In contrast, a previous study by Suslu et al. [22] found greater myelin degeneration at 28 days in animals treated with systemic dexamethasone compared to those receiving perineural dexamethasone. Yao et al. [25] examined the impact of dexamethasone pellets in a model of transected hypoglossal nerve, reporting a higher degree of reprojected neurons at 28 days compared to controls. However, this model differs substantially from clinical scenarios in regional anesthesia, as the hypoglossal nerve is a purely motor cranial nerve originating from the brainstem and exiting via the hypoglossal canal. In contrast, peripheral nerves typically arise from the spinal cord and contain mixed motor, sensory and sometimes autonomic fibers, and they also have a higher degree of myelination [28].

The effect of delayed treatment (4 weeks since the initial nerve injury) with dexamethasone on a model of nerve injury was investigated by Wang et al. [24]. No statistically significant differences in demyelination were observed among groups treated with either low or high doses of perineural dexamethasone and sham controls.

Accordingly Bastos et al. [16] investigated the effects of dexamethasone-loaded biodegradable implants in a sciatic nerve injury model induced by nerve ligation. They found that implant application at the time of injury reduced p65/β-actin expression in the L4–L5 dorsal root ganglia (DRG) by approximately two-thirds compared to delayed application 12 days post-injury. The p65/β-actin ratio serves as an indicator of NF-κB (nuclear factor kappa-light-chain-enhancer of activated B cells) activation, a transcription factor critically involved in the DRG’s modulation of pain signaling and neuropathic pain development.

In addition, a previous study by Matsuda et al. [20] demonstrated that pain stimuli increase nitric oxide expression in the DRG. This elevated nitric oxide level was reduced by a single-shot ropivacaine block and further decreased by the addition of perineural dexamethasone. However, even if tested, the study did not report results for mice receiving systemic dexamethasone, making it unclear whether the observed effects of dexamethasone were due to local or systemic action.

## Discussion

Based on available in vitro and in vivo evidence, perineural dexamethasone appears to have a generally favorable safety profile and potential neuroprotective effects when used as an adjuvant to local anesthetics. In vitro data suggest that while dexamethasone alone is not neurotoxic, its combination with local anesthetics may modestly increase cytotoxicity—though not significantly—at clinically relevant doses. However, higher concentrations, especially those containing sulfites, can lead to neural cell death, highlighting the importance of formulation and dose.

Important considerations have to be made regarding the in vitro model we have retrieved: neuroblastoma cell lines and DRG cells. Both are frequently used models in neurotoxicity and neuroprotection research. In particular, neuroblastoma cell lines are used due to their neural crest origin and capacity to undergo neuronal differentiation. These cells are derived from the sympathetic nervous system and thus share developmental lineage with peripheral neurons and can exhibit phenotypic characteristics of mature neurons, including neurite outgrowth, expression of neuronal markers and electrical excitability [29].

However, neuroblastoma cells are tumor-derived and immortalized, and thus differ significantly from primary peripheral neurons in key aspects such as receptor expression, ion channel composition, and metabolic activity. Their proliferative nature and altered genomic profile may limit the generalizability of findings to physiologically normal peripheral nerve cells [30].

For these reasons, neuroblastoma cell models should ideally be used in conjunction with primary DRG neurons, which are considered the gold standard for in vitro modeling of peripheral nerve function and injury. DRG neurons more accurately replicate the physiological and morphological characteristics of peripheral sensory neurons, including appropriate ion channel expression and susceptibility to neurotoxic stimuli [31]. While primary DRG cultures are technically demanding and limited by species and availability, they provide critical biological relevance that complements the ease and scalability of neuroblastoma-based assays.

In vivo studies reinforce the relative safety of perineural dexamethasone. Most histological analyses reported no significant differences in nerve inflammation, axon degeneration or demyelination compared to controls, particularly beyond 7 days. Notably, early signs of reduced inflammation and apoptosis, as well as preserved Schwann cell function, have been observed with perineural (but not systemic) administration [15]. Although a reduction in blood flow was documented following perineural dexamethasone administration [21], the magnitude of this decrease remained well below the established threshold required to induce ischemic nerve damage in healthy models [27]. However, this finding should be interpreted with caution in clinical practice, particularly in patients with preexisting neuropathies such as diabetic neuropathy. These individuals often have compromised microvascular perfusion and reduced nerve resilience, potentially making them more susceptible to even modest reductions in blood flow. As such, while the risk of ischemia appears minimal in otherwise healthy nerves, the same may not hold true in vulnerable populations. Therefore, a careful risk–benefit assessment should be undertaken when considering the use of perineural dexamethasone in patients with underlying neuropathic conditions, weighing its anti-inflammatory and neuroprotective potential against the theoretical risk of exacerbating ischemic injury.

Furthermore, in nerve injury models, dexamethasone attenuated molecular markers of apoptosis and inflammation (e.g. caspase-3, NF-κB), particularly when administered immediately after injury. Its delayed application, however, showed limited benefit.

Our systematic review poses the pillars for future research; in particular, we believe that several avenues of investigation are warranted such as long-term safety evaluations as existing studies generally assess histological outcomes over relatively short time frames while extended observational periods are needed to rule out delayed neurotoxicity, demyelination or other chronic effects; dose–response and formulation studies as standardized testing of preservative-free versus sulfite-containing formulations at clinically relevant concentrations would clarify formulation-specific risks; vulnerable populations as one of the most intriguing aspect of our findings is the potential effect in these populations, experimental models simulating diabetic neuropathy or aging-related microvascular dysfunction could help assess whether observed effects are magnified in at-risk patient groups.

## Conclusions

In vivo and in vitro models support the safety of low-dose, preservative-free perineural dexamethasone. However, based on the findings and considering the high risk of bias in the available studies, the authors recommend caution in its clinical use. Specifically, the use of high dexamethasone doses, formulations containing additives, and the use in patients with evident or latent neuropathies could be potentially risky. This is especially relevant considering the limited additional clinical benefit of perineural dexamethasone compared to systemic administration.

## Supplementary Information

Supplementary Material 1.

## Data Availability

No datasets were generated or analysed during the current study.
