# Supplementary material for: Perineural dexamethasone: neurotoxicity or neuroprotection? A systematic review of preclinical evidence
Source: J Anesth Analg Crit Care. 2025 Aug 6;5:50. doi: 10.1186/s44158-025-00271-w (PMC12329900; doi:10.1186/s44158-025-00271-w)
Supplement: Supplementary file 1 — Supplementary Material 1. [file 44158_2025_271_MOESM1_ESM.pdf]

Perineural Dexamethasone: Neurotoxicity or Neuroprotection? A Systematic Review of Preclinical Evidence

– – –

Supplementary Digital Content

1) Search strategy p. 2
2) Excluded articles with reasons p. 3
3) SYRCLE Risk of bias table for in-vivo studies p. 4

1) Search Strategy

Pubmed
("Dexamethasone"[Mesh] OR dexamethasone[tiab]) AND ("Peripheral Nerves"[Mesh] OR perineural[tiab] OR "nerve block"[tiab] OR "regional anesthesia"[tiab]) AND ("Neurotoxicity Syndromes"[Mesh] OR neurotoxicity[tiab] OR "nerve injury"[tiab] OR "nerve damage"[tiab] OR "neural damage"[tiab] OR "histological"[tiab] OR "electrophysiological"[tiab])
**Scopus**
(TITLE-ABS-KEY (dexamethasone)) AND (TITLE-ABS-KEY (perineural OR "nerve block" OR "regional anesthesia")) AND (TITLE-ABS-KEY (neurotoxicity OR "nerve injury" OR "nerve damage" OR "neural damage" OR histological OR electrophysiological))
**Embase**
1. dexamethasone/ OR dexamethasone.tw.<br>2. perineural.tw. OR nerve block.tw. OR regional anesthesia.tw. OR nerve block/ OR regional anesthesia/<br>3. neurotoxicity.tw. OR nerve injury.tw. OR nerve damage.tw. OR neural damage.tw. OR histological.tw. OR electrophysiological.tw. OR neurotoxicity/ OR nervous system injury/<br>4. 1 AND 2 AND 3
**Cochrane CENTRAL**
(dexamethasone):ti,ab,kw<br>AND<br>(perineural OR "nerve block" OR "regional anesthesia"):ti,ab,kw<br>AND<br>(neurotoxicity OR "nerve injury" OR "nerve damage" OR "neural damage" OR histological OR electrophysiological):ti,ab,kw

2) Excluded full text with reasons

1. Lee JB, Choi SS, Ahn EH, Hahm KD, Suh JH, Leem JG, Shin JW. Effect of perioperative perineural injection of dexamethasone and bupivacaine on a rat spared nerve injury model. Korean J Pain. 2010 Sep;23(3):166-71. doi: 10.3344/kjp.2010.23.3.166. Epub 2010 Aug 26. PMID: 20830261; PMCID: PMC2935977.

Investigated the analgesic effect of perineural dexamethasone without providing any information on a possible neurotoxic or neuroprotective effect

2. Williams BA, Butt MT, Zeller JR, Coffee S, Pippi MA. Multimodal perineural analgesia with combined bupivacaine-clonidine-buprenorphine-dexamethasone: safe in vivo and chemically compatible in solution. Pain Med. 2015 Jan;16(1):186-98. doi: 10.1111/pme.12592. Epub 2014 Oct 23. PMID: 25339320.

This study analyzes Dexamethasone in a formulation that also comprises Clonidine and Buprenorphine.

3. Shichor I, Shomron N, Lawlor MW, Bae SA, Zoldan J, Langer R, Kohane DS. Toxicogenomic analysis of a sustained release local anesthetic delivery system. Biomaterials. 2012 May;33(13):3586-93. doi: 10.1016/j.biomaterials.2012.01.043. Epub 2012 Feb 16. PMID: 22341215; PMCID: PMC3288387.

In this paper dexamethasone was not alone or together with only local anesthesia

4. Mohamed RMSM, Ahmad Ahmad E, Amin DM, Abdo SA, Ibrahim IAAE, Mahmoud MF, Abdelaal S. Adrenergic receptors blockade alleviates dexamethasone-induced neurotoxicity in adult male Wistar rats: Distinct effects on β-arrestin2 expression and molecular markers of neural injury. Daru. 2024 Jun;32(1):97-108. doi: 10.1007/s40199-023-00490-y. Epub 2023 Nov 15. PMID: 37966585; PMCID: PMC11087427.

This study analyzes the effect of high dose of systemic dexamethasone on the brain

5. Johansson A, Hao J, Sjölund B. Local corticosteroid application blocks transmission in normal nociceptive C-fibres. Acta Anaesthesiol Scand. 1990 Jul;34(5):335-8. doi: 10.1111/j.1399-6576.1990.tb03097.x. PMID: 2167604.

This study analyzes the transmission in C-fibers after Dexamethasone application

6. Mackinnon SE, Hudson AR, Gentili F, Kline DG, Hunter D. Peripheral nerve injection injury with steroid agents. Plast Reconstr Surg. 1982 Mar;69(3):482-90. doi: 10.1097/00006534-198203000-00014. PMID: 7063571.

The full-text downloadable from the publisher site is a two page pdf with only the first and the last page. Further attempts to retrieve the full-text were unsuccessful

3) SYRCLE Risk of bias table for in-vivo studies

	An (2015)	Bastos (2019)	Çömez (2020)	Ferré (2020)	Marty (2018)	Matsuda (2022)	Suslu (2013)	Wang (2015)	Yao (1995)	Shishido (2002)	Wang (2011)
Sequence generation	Not stated	Not stated	Not stated	Stated as randomized	Simple randomization	Not stated	Not stated	Randomly assigned	Not stated	Not stated	Not stated
Baseline characteristics	Not stated	Wight, gender	Not stated	Not stated	Not stated	Not stated	Not stated	Not stated	Not stated	Not stated	Not stated
Allocation concealment	Not stated	Not stated	Not stated	Drugs were prepared by a not involved researcher	Not stated	Not stated	Not stated	Not stated	Not stated	Not stated	Not stated
Random housing	No, animals returned to their cage	Not stated	Not stated	Not stated	Not stated	Not stated	Not stated	Not stated	Not stated	Not stated	Not stated
Blinding	Not stated	Behavioural assessors described as blind	Not stated	Assessors described as blind	Assessors described as blind	Not stated	Not stated	Not stated	Not stated	Not stated	Not stated
Random outcome assessment	All animals were assessed	Not stated	Not stated	Animals were selected at random	Not stated if they were sacrified at different timepoints in a randomized order	Not stated	Not stated	Not stated	Not stated	Not stated	Not stated
Blinding	Not stated	Behavioural assessors described as blind	Outcome assessor was blind	Outcome assessor was blind	Outcome assessor was blind	Outcome assessor was blind	Outcome assessor was blind	Outcome assessor was blind	Not stated	Not stated	Not stated
Incomplete outcome data	No incomplete outcome data	Probably missing data for many outcomes (numbers and time are not clearly explained)	No incomplete outcome data	Data was almost complete (one specimen not included in the histopatological analysis)	Data was almost complete (one specimen not included in the histopatological analysis)	Some outcomes not reported (for example regarding sytemic dexamethasone)	No incomplete outcome data	No incomplete outcome data	No incomplete outcome data	No incomplete outcome data	No incomplete outcome data
Selective outcome reporting	No study protocol available	No study protocol available	No study protocol available	No study protocol available	No study protocol available	No study protocol available	No study protocol available	No study protocol available	No study protocol available	No study protocol available	No study protocol available
Other sources of bias	No other source of bias	Number of animals used in the experiment and the time to sacrifice was not specified	No other source of bias	No other source of bias	No other source of bias	No other source of bias	No other source of bias	No other source of bias	No other source of bias	No other source of bias	No other source of bias
